# Patterns and drivers for wetland connections in the Prairie Pothole Region, United States

**DOI:** 10.1007/s11273-016-9516-9

**Published:** 2016-11-19

**Authors:** Melanie K. Vanderhoof, Jay R. Christensen, Laurie C. Alexander

**Affiliations:** 1 Geosciences and Environmental Change Science Center, U.S. Geological Survey, DFC, MS980, 25046, Lakewood, CO 80225 USA; 2 Office of Research and Development, National Exposure Research Laboratory, Environmental Science Division, U.S. Environmental Protection Agency, 944 E. Harmon Ave.,, Las Vegas, NV 89119 USA; 3 Office of Research and Development, National Center for Environmental Assessment, U.S. Environmental Protection Agency, 1200 Pennsylvania Ave. NW (8623-P), Washington, DC 20460 USA

**Keywords:** Wetlands, Prairie Pothole Region, Connectivity, Network, Landsat, Wetland regulation

## Abstract

Ecosystem function in rivers, lakes and coastal waters depends on the functioning of upstream aquatic ecosystems, necessitating an improved understanding of watershed-scale interactions including variable surface-water flows between wetlands and streams. As surface water in the Prairie Pothole Region expands in wet years, surface-water connections occur between many depressional wetlands and streams. Minimal research has explored the spatial patterns and drivers for the abundance of these connections, despite their potential to inform resource management and regulatory programs including the U.S. Clean Water Act. In this study, wetlands were identified that did not intersect the stream network, but were shown with Landsat images (1990–2011) to become merged with the stream network as surface water expanded. Wetlands were found to spill into or consolidate with other wetlands within both small (2–10 wetlands) and large (>100 wetlands) wetland clusters, eventually intersecting a stream channel, most often via a riparian wetland. These surface-water connections occurred over a wide range of wetland distances from streams (averaging 90–1400 m in different ecoregions). Differences in the spatial abundance of wetlands that show a variable surface-water connection to a stream were best explained by smaller wetland-to-wetland distances, greater wetland abundance, and maximum surface-water extent. This analysis demonstrated that wetland arrangement and surface water expansion are important mechanisms for depressional wetlands to connect to streams and provides a first step to understanding the frequency and abundance of these surface-water connections across the Prairie Pothole Region.

## Introduction

Depressional wetlands provide critical hydrological services including storing precipitation and hydrologic inflows (Winter and Rosenberry 1998), which reduces peak stream flows and potential downstream flooding (Vining 2002; Yang et al. 2010). As depressional wetlands are typically non-channel connected (Tiner 2003), surface-water connections with other water bodies are usually not continuous. Under wet conditions, however, many of these wetlands cyclically or episodically exchange or contribute water to other wetlands, open waters, and/or streams through temporary overland or shallow groundwater flows, unmapped ditches or channels, and/or the merging of wetland waters in low relief areas (Rains et al. 2006, 2008; Cook and Hauer 2007; Sass and Creed 2008; Kahara et al. 2009; Wilcox et al. 2011; McCauley et al. 2015). During flood events or wet periods, depressional wetlands that become connected to streams or subsumed by lakes exchange water and materials, but may experience the temporary loss of wetland function until water levels recede (Junk et al. 1989; Galat et al. 1998; Mortsch 1998). Understanding landscape drivers for the abundance of wetlands that cyclically or variably contribute water to streams is important for accurately predicting stream flow, particularly during high flow events (Vining 2002; Yang et al. 2010), as well as informing the process to determine the jurisdictional status of wetlands in compliance with the U.S. Clean Water Act. Yet, relatively little research into variable surface-water connections, in particular landscape patterns and drivers of such connections, has been done (U.S. EPA 2015).

The Prairie Pothole Region (PPR), in north-central North America, is known for its high density of depressional wetlands (Sorensen et al. 1998). Substantial variation in surface-water extent in response to climate is well-documented within the region (Beeri and Phillips 2007; Zhang et al. 2009; Niemuth et al. 2010; Huang et al. 2011a; Liu and Schwartz 2011). Changes to surface waters can result in variable wetland-to-wetland (Winter and Rosenberry 1998; Kahara et al. 2009) and wetland-to-stream connectivity (Leibowitz and Vining 2003; Sass and Creed 2008; Vanderhoof et al. 2016). Minimal research, however has sought to explain the abundance of wetlands that show variable surface-water connections, or understand at a landscape-scale how these wetlands consolidate and become connected to streams.

The primary metric used to identify wetlands that may lack a surface-water connection has been landscape position. This metric has been quantified using variables such as feature density, area, proximity, and cohesion, (Kahara et al. 2009) or distance to stream (Tiner 2003; Lang et al. 2012; Lane and D’Amico 2016). However, most of these efforts have been theoretical and have not related dynamic spatial variables, such as distance, to changes in actual surface-water extent. In addition to landscape position, the probability of connectivity for an individual wetland can also be expected to depend on climate, which influences the magnitude, frequency and duration of water inputs (Phillips et al. 2011); topography, which influences the capacity for surface-water expansion (Rover et al. 2011; Shaw et al. 2013); and anthropogenic drainage (i.e., ditches and tile drainage), which modifies topographical flows of surface water (McCauley et al. 2015). Although existing research has contributed to our ability to predict fill-and-spill on the scale of individual wetlands (Huang et al. 2011b) and in small, heavily instrumented watersheds (Shaw et al. 2012; Spence and Phillips 2014), efforts to understand the abundance of surface-water connections on a landscape scale have been minimal.

In this study, we identified wetlands that became connected to a stream in at least one of 16–17 Landsat images (acquired between 1990 and 2011) as surface-water extent expanded. Vanderhoof et al. (2016) quantified and examined temporal variability from drought to deluge, in surface-water connections between wetlands and streams in the PPR. In this study we eliminated the temporal aspect and instead investigated spatial landscape patterns related to the abundance of variable surface-water connections, exploring specifically, (1) the spatial mechanism and wetland cluster size through which wetlands merge with streams in wet years, (2) patterns in distance metrics for variably connected wetlands in relation to streams, and (3) landscape variables influencing the spatial abundance of these variably connected wetlands. This study builds upon previous work by evaluating the performance of simple distance metrics and investigating the use of landscape-scale parameters to explain variation in the abundance of surface-water connections within a region. Improved understanding of the drivers for the abundance of wetlands that cyclically or episodically contribute water to streams has implications for hydrological predictions as well as water resource management and policy.

## Methods

The per-pixel fraction water was derived for Landsat imagery using a partial unmixing algorithm. Aerial imagery from multiple dates was then used to (1) threshold the continuous fraction water into water and upland cover types, and (2) validate the Landsat-derived maps of surface-water extent using a random point analysis. The Landsat-derived surface water maps were overlaid with wetland and stream reference datasets to identify a class of variabily connected (VC) wetlands, i.e., wetlands that became connected to a stream in at least one of the images as surface-water extent expanded. Wetland cluster size, mechanism of connection to a stream, and distance to stream for this class of wetlands were compared across six ecoregions. Lastly, the relative importance of explanatory variables was assessed to identify landscape factors influencing the abundance of wetlands in this class across the study area.

### Study area

**Fig. 1:**
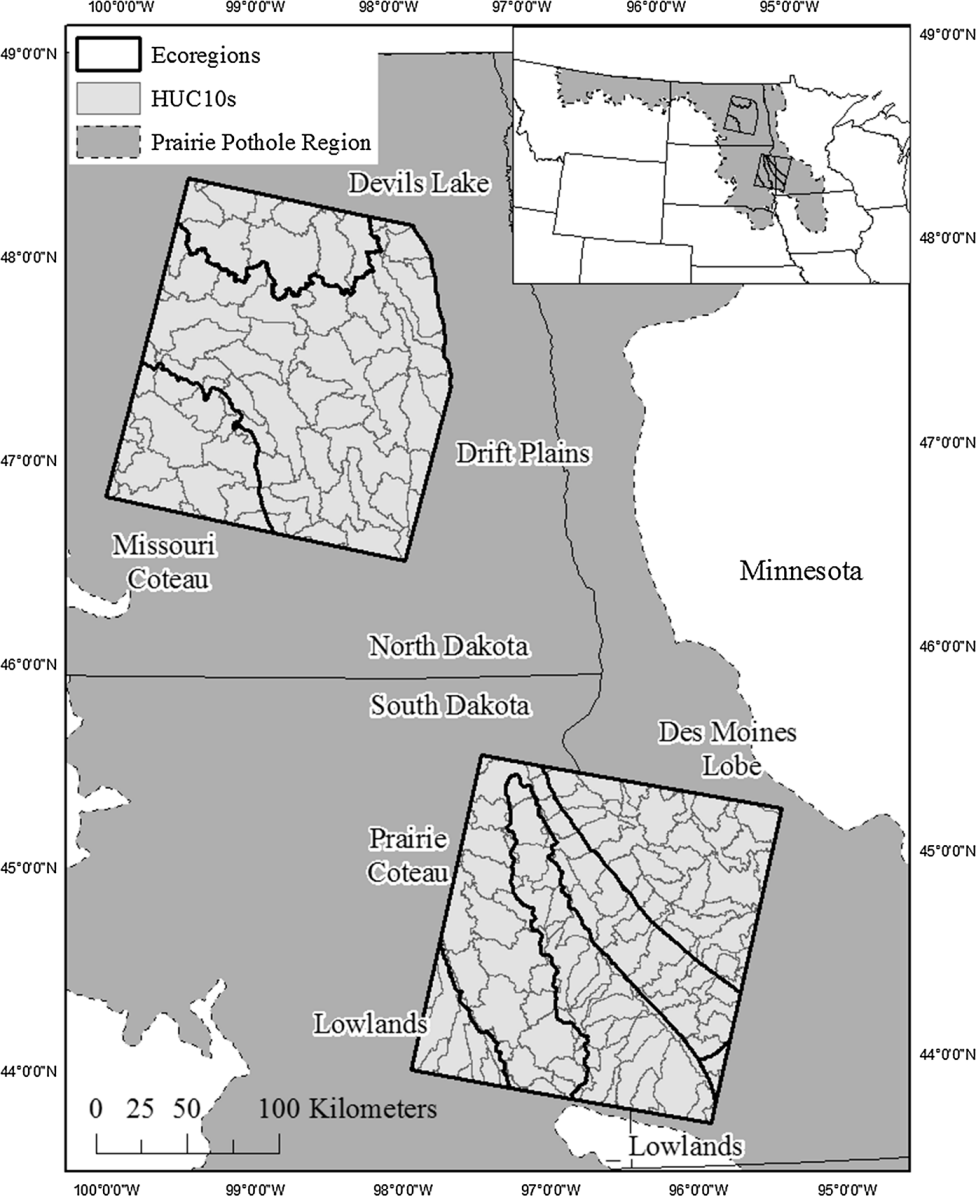
The distribution of the units of analysis including ecoregions (n = 6) and HUC10 s (n = 155) within the two Landsat path/row extents (p31r27, northern path/row, p29r29, southern path/row)

Ecoregions (n = 6) (Omernik and Griffith 2014) and 10-digit hydrological units (HUC10) (n = 155) (Seaber et al. 1987) were used as the units of analysis across two, non-adjacent, Landsat path/rows [p29r29 (southern path/row) and p31r27 (northern path/row)] within the United States portion of the PPR (Fig. 1). The ecoregions included in this study represent a diversity of physiographic regions (Table 1) but were not intended to capture all possible variation that might occur in the PPR from Montana east to the Red River Valley, south into Iowa and north into Alberta and Saskatchewan. Ecoregion extent was modified from the U.S. Environmental Protection Agency Level IV Ecoregion definitions (Omernik and Griffith 2014), and included (1) Lowlands (included the Big Sioux Basin, James River Lowland and Loess Prairie ecoregions),(2) Des Moines Lobe (included the Minnesota River Prairie and Tewaukon/Big Stone Stagnation Morraine ecoregion), (3) Prairie Coteau, (4) Missouri Coteau, (5) Drift Plains, and (6) Devils Lake (Fig. 1; Table 1). HUC10 s were used as the unit of analysis (Seaber et al. 1987) for the modeling component of the study (Fig. 1). Land cover across the study area is dominated by cultivated crops (56%), hay/pasture (13%) and herbaceous vegetation (14%) (Homer et al. 2015). Average summer (June–August, 20.5 °C) and winter (December–February, −8.6 °C) temperatures (1981–2010) are similar across the study area, while mean annual precipitation (1981–2010) is lower in the northern path/row (496 mm yr^−1^, 37 mm winter, 119 mm spring, 228 mm summer, and 112 mm fall^)^, relative to the southern path/row (649 mm yr^−1^, 40 mm winter, 175 mm spring, 273 mm summer, 161 mm fall) (NOAA NCDC 2014).

**Table 1:** Characteristics of ecoregions as derived from National Wetland Inventory (NWI) and National Hydrography Dataset (NHD) datasets

Ecoregion	Size (ha)	Annual Precipitation (1981–2010) Normal (mm)	NHD stream density (m ha^−1^)	NWI wetland area (m^2^ ha^−1^)	NWI wetland density (# per ha)	Non-channel connected NWI wetlands (%)	Non-channel connected NWI wetland area (%)
Lowland	887,232	653	13.4	485.3	0.06	80.0	40.0
Des Moines Lobe	824,612	648	10.1	616.6	0.02	83.4	31.6
Prairie Coteau	1215,080	646	9.7	1184.8	0.07	88.4	41.1
Missouri Coteau	524,552	484	3.0	1369.7	0.11	96.9	75.1
Drift Plains	1892,710	507	5.1	893.6	0.12	96.7	75.5
Devils Lake	443,718	507	1.8	2092.0	0.18	99.3	51.9

Non-channel connected NWI wetlands are NWI wetlands that do not intersect the NHD stream buffer

### Image processing

Landsat images were selected to coincide with snow-free conditions and restricted to images with <10% cloud cover. Cloud-free spring images were utilized when available to capture seasonal peaks in surface-water extent post-snowmelt. Seventeen and sixteen images were included for the southern and northern path/rows, respectively. The images included conditions characterized as the 99% wettest by the monthly Palmer Hydrological Drought Index (PHDI), calculated from precipitation and temperature station data and interpolated at 5 km (NOAA NCDC 2014) (Table 2). Images were atmospherically corrected and converted to surface reflectance values using the Landsat Ecosystem Disturbance Adaptive Processing System (Masek et al. 2006). The Matched Filtering algorithm, a partial unmixing method in the ENVI software package (Exelis Visual Information Solutions, Inc, Herndon, Va) was used to produce the per pixel water fraction and classify these outputs into water and upland cover classes (Turin 1960; Frohn et al. 2012). Error was reduced by applying a minimum noise fraction transformation to reduce noise (Green et al. 1988), linearly stretching output values to maximize category separability, and masking out impervious surfaces, defined as low, medium and high density development land cover types to reduce false positives (Homer et al. 2015).

**Table 2:** Landsat Thematic Mapper (TM) images utilized in the analysis and corresponding monthly Palmer Hydrological Drought Index (PHDI) values

Path/row	Landsat TM image	PHDI	Path/row	Landsat TM image	PHDI
p29r29	10-May-90	−3.55	p31r27	9-Jun-90	−4.12
p29r29	13-May-91	−0.69	p31r27	12-Jun-91	−2.45
p29r29	15-May-92	−1.15	p31r27	27-Apr-92	−1.93
p29r29	23-Sep-93	6.86	p31r27	26-Oct-94	7.03
p29r29	15-Oct-95	6.37	p31r27	27-Sep-95	5.97
p29r29	14-Jun-97	4.02	p31r27	14-Jul-97	−0.09
p29r29	30-Apr-98	2.77	p31r27	1-May-99	2.01
p29r29	*8-May-01	4.47	p31r27	9-Jul-01	4.46
p29r29	19-Nov-02	−1.69	p31r27	5-Oct-04	4.38
p29r29	28-Apr-03	−2.01	p31r27	*18-Jun-05	1.45
p29r29	1-Apr-05	3.15	p31r27	9-Sep-06	−2.91
p29r29	4-Apr-06	4.2	p31r27	12-Sep-07	2.41
p29r29	13-Oct-06	2.3	p31r27	1-Sep-09	3.28
p29r29	15-Apr-10	5.43	p31r27	6-Oct-10	6.43
p29r29	8-Oct-10	9.63	p31r27	5-Jul-11	6.61
p29r29	*5-Jun-11	8.37	p31r27	*11-Sep-11	8.92
p29r29	11-Oct-11	5.88			

* Dates defined as deluge conditions

Surface-water extent was defined as saturated soil (i.e., visibly wet soil often adjacent to open water features) or wetter (i.e., inundated or open water). The water-upland threshold was derived by distinguishing the mean fraction water for saturated soil (239 total points) from the mean fraction water for upland photosynthetic vegetation (183 total points) using data points visually classified from 1-m National Agricultural Imagery Program (NAIP) imagery from three dates per Landsat path/row (April 30, 2004, October 13, 2006 and October 8, 2010 for p29r29; July 1, 2004, October 5, 2004, and September 9, 2006 for p31r27). The derived threshold for water (>0.26) was meant to include mixed pixels (e.g. shallow water or shallow sub-surface flow, wetland edges, and vegetated water) (e.g., Sass and Creed 2008). However, most small (~3–10 m wide) channel-swale features (261 total points), which were also tested, represented a minor fraction of the Landsat pixel and were, on average, spectrally indistinguishable from upland photosynthetic vegetation and therefore unidentifiable. Differences in the fraction of water were larger between cover categories than between dates, so that the threshold was applied across all dates and both path/rows. The outputs were surface-water extent maps.

### Validation analysis

The surface-water extent maps were validated using an independent data source, 1 m resolution NAIP imagery in a (1) random point analysis; and (2) minimum wetland size detection analysis. In the random point analysis, 1500 points (250 per Landsat path/row, same three NAIP dates per path/row as threshold analysis) were randomly selected. NAIP imagery was limited to images collected at dates similar to the Landsat imagery to minimize differences in surface-water extent between the two sources. Outcomes (surface water vs. upland) were visually compared between NAIP and the Landsat derived surface-water maps. Upland was defined as any pixel that did not meet the fraction of water threshold. Producer accuracy was the probability that a Landsat pixel was classified as surface water given that surface water was indicated by the NAIP imagery, while user accuracy was the probability that the NAIP imagery showed surface water being present given a Landsat pixel classified as surface water. Overall accuracy (percent of all points correctly classified) was 96.5%. The producer accuracy for surface water was 94.6%, while the user accuracy for surface water was 88.4% (Table 3). A threshold that allowed more mixed pixels or small wetland features, to be identified, produced a high producer accuracy (i.e., low omission error), but in turn reduced the user accuracy (i.e., introduced errors of commission) due to (1) limited confusion with the high leaf water content in dense agricultural fields in the Des Moines Lobe ecoregion, and (2) mixed-pixel or scale-related errors (e.g., NAIP image points located at the edge of features which resulted in a mixed Landsat pixel). To determine the minimum wetland size that was reliably detected, we randomly selected a total of 421 National Wetland Inventory (NWI) wetlands from the NAIP imagery that were <0.1 ha to 1 ha in size. Wetlands were selected that (1) were individual wetland features (i.e., not part of a larger wetland cluster), and (2) showed at least some open water. Seventy nine percent of wetlands larger than 0.2 ha were reliably detected.

**Table 3:** Accuracy assessment for the surface water extent maps, comparing Landsat derived surface water and upland classification maps, to 1 m National Agricultural Imagery Program (NAIP) aerial imagery

Map accuracy	NAIP–Wetland	NAIP–Upland	Total points
Landsat—Wetland	283	37	320
Landsat—Upland	16	1164	1180
Total	299	1201	1500
Producer accuracy for wetland (%)	94.6		
User accuracy for wetland (%)	88.4		
Overall accuracy (%)	96.5		
Kappa statistic	0.9		

### Landscape analysis

**Fig. 2:**
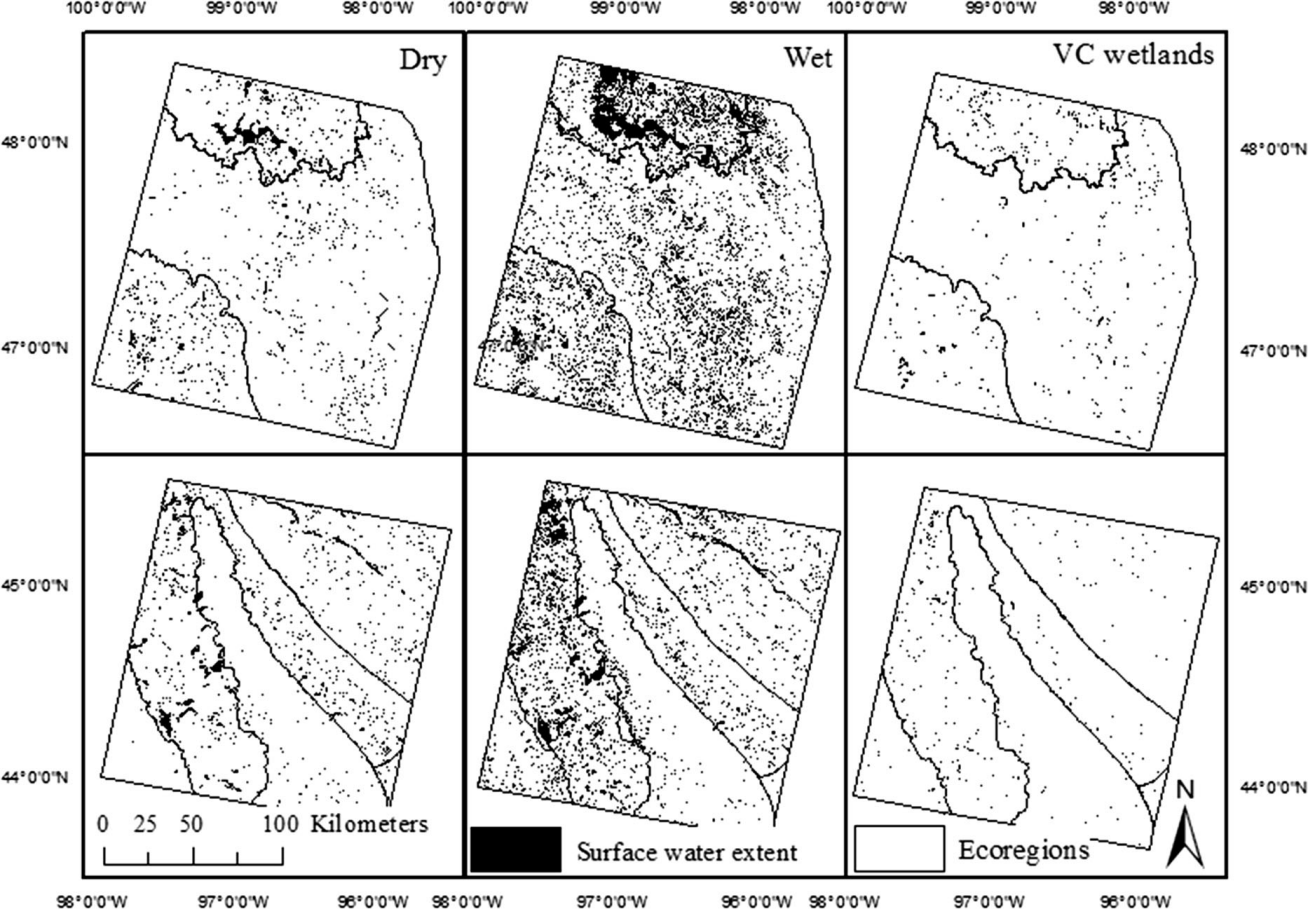
Patterns of water cover (saturated) for dry (*Pr*(0.06) Cumulative distribution function (CDF)) Palmer Hydrological Drought Index (PHDI) (spring 1990) (*left*) and wet (*P*r(0.99) CDF PHDI) (spring 2011) (*middle*) conditions for p31r27 (*top row*) and p29r29 (*bottom row*). The spatial distribution of variably connected (VC) National Wetland Inventory (NWI) wetlands is shown for both path/rows (*right*). *note* Most small wetlands are NOT visible due to the scale of the images

The NWI dataset (U.S. Fish and Wildlife Service, 2010) was used as the reference wetland dataset and was designed to represent wetland extent under “average” hydrological conditions (USFWS 2010). Stream occurrence was defined by the high resolution National Hydrography Dataset (NHD) (1:24,000) (USGS 2013). A stream buffer was applied (± 14 m) to account for the nationally reported digital accuracy of the lateral location of stream features within this dataset (USGS 2000). The stream/river NHDArea polygons were included to account for channel width. The NWI and NHD were used with additional datasets to derive landscape variables for assessing landscape influence on the abundance of surface-water connections. *Lake count* and *total lake area* were defined as the subset of NWI polygons classified as lacustrine (0.4% of all NWI polygons in the study area). *Maximum surface*-*water extent* was derived from Landsat image showing the greatest surface-water extent (spring 2011 for both path/rows) (Fig. 2). *Change in surface*-*water extent* was derived by subtracting the total surface-water extent from the driest image (spring 1990 for p29r29, spring 1991 for p31r27) (ha), from the surface-water extent in the wettest image (spring 2011) (ha) (Fig. 2). Surface topography, which can influence the capacity for surface water to expand, was quantified as the (1) *elevation coefficient of variation* across each HUC10 (Ascione et al. 2008), as well as the (2) Melton ruggedness number, which is calculated as the maximum elevation minus the minimum elevation divided by the HUC10 area (Melton 1965), using the USGS National Elevation Dataset (NED) 10 m resolution (Gesch et al. 2002). Lastly, to account for anthropogenic modifications to drainage systems, the *percent land cover artificially drained* was estimated as the percent of each HUC with collocated row crop cover type (derived from the National Land Cover Database (NLCD) 2006) and very poorly drained or poorly drained soils as defined by the National Resources Conservation Service’s SSURGO database (Christensen et al. 2013). The distribution of values within the explanatory variables are shown in Table 4.

**Table 4:** Explanatory variables and units considered by linear regression models

Variables	Units	Range	25th percentile	50th percentile	75th percentile	Source
Stream density	km ha^−1^	0.0011–0.021	0.0037	0.0074	0.011	High Res. National Hydrograph Dataset (NHD) (USGS 2013)
Wetland to stream Euclidean distance	m	55.58–3195.32	306.73	520.64	1215.54	High Res. NHD (USGS 2013)
Lake abundance (count)	no ha^−1^	0–0.0052	0.00,059	0.0012	0.0019	National Wetland Inventory (USFWS 2010)
Lake areal abundance	ha ha^−1^	0–0.92	0.023	0.054	0.096	National Wetland Inventory (USFWS 2010)
Maximum surface water extent	ha ha^−1^	0.0091–0.41	0.070	0.10	0.19	Landsat images
Change in surface water extent	ha ha^−1^	0.00,031–0.37	0.037	0.067	0.13	Landsat images
Total wetland density	no ha^−1^	0.0059–0.27	0.032	0.076	0.12	National Wetland Inventory (USFWS 2010)
Total wetland areal abundance	ha ha^−1^	0.0032–0.26	0.040	0.077	0.12	National Wetland Inventory (USFWS 2010)
Wetland to wetland Euclidean distance	m	49.02–351.84	69.32	88.13	128.30	National Wetland Inventory (USFWS 2010)
Percent drained by anthropogenic means	%	0.15–60.06	1.85	3.85	16.78	National Land Cover Database and Soil Survey Geographic Database (Christensen et al. 2013)
Elevation coefficient of variation	m	5E−08–0.23	0.021	0.033	0.053	National Elevation Dataset (NED) 10 m Digital Elevation Model (DEM) (Gesch et al. 2002)
Melton ruggedness number	m km^2^	0.17–3.43	0.38	0.54	0.83	NED 10 m DEM ((Zmax–Zmin)/area) (Gesch et al. 2002)

Range and percentiles are provided to show distribution of values for each variable across all hydrological units (HUC10s)

### Wetland connectivity classification and mechanism for connection

**Fig. 3:**
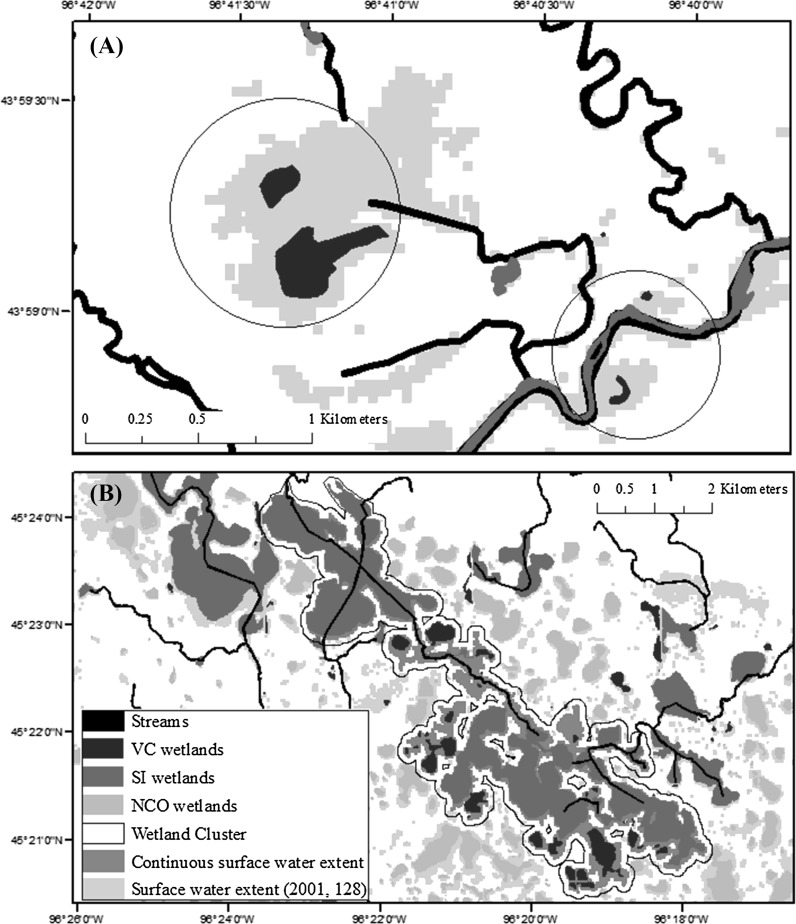
Wetlands that showed a variable connection to streams (VC wetlands) occurred in clusters of varying size as shown by **a** VC wetlands connecting to a tributary of the Big Sioux River (*left circle*) and individually to the Big Sioux River (*right circle*), and **b** 32 VC wetlands connecting within a continuous cluster to a tributary of the Minnesota River

“Surface-water connection” is used as a general term indicating multiple mechanisms, including wetland fill-and-spill, merging and subsuming of wetlands by lakes or other wetlands and stream overbank flow. In using this term, we make no assumption about shifts or loss of wetland function that co-occur with surface-water expansion. The NWI wetlands were separated into three classes for this analysis, (1) wetlands which directly intersected the NHD stream layer (including stream polygons and buffer) and were considered to show a semi-permanent or permanent connection to a stream (SI or stream-intersect wetlands), (2) NWI wetlands which did not intersect the stream layer, but intersected a stream-connected patch of surface water, as mapped by Landsat in at least one of the Landsat images (VC or variably connected wetlands) (Fig. 2), and (3) NWI wetlands for which no stream connection was observed in any of the Landsat images (NCO or no connection observed wetlands) (Fig. 3). It is important to note that occasional or even frequent surface-water stream connections may also occur for wetlands included in the third category (NCO). Landsat imagery can be expected to bias the analysis towards surface-water connections that occur through the expansion of relatively broad features such as river overflow into floodplains, or features merging or being subsumed from increases in water level or filling and spilling. Cyclical or episodic linear connections (e.g., ephemeral channels, swales, ditches) that connect some waters (e.g., Tromp-van Meerveld and McDonnell 2006) are often not well documented by NHD, which has been shown to inconsistently map such features (Lang et al. 2012; Fritz et al. 2013) and are difficult to detect with Landsat. Although this approach to observing all VC wetlands is limited due to a low probability of detecting narrow and/or short duration (hours to days) connections, it allows us to identify regionally relevant parameters that may influence the abundance of such wetlands in the PPR.

The mechanism through which VC wetlands connect to streams was also investigated. Most of the VC wetlands merged with streams only under deluge or very wet conditions. Therefore, to derive the distribution of VC wetlands by wetland cluster size (or a complex of surficially-connected wetlands or consolidated wetlands), the number of VC wetlands co-occurring within a single Landsat-derived surface-water polygon was quantified using the two wettest (greatest percent area classified as inundated and saturated) images for each path/row (spring 2005 and 2011 for p31r27, spring 2001 and 2011 for p29r29) (Fig. 3). The mechanism of connection for VC wetlands was further classified as, (1) connecting through a wetland cluster containing a SI wetland, (2) connecting through individual expansion of a wetland and/or stream, or (3) connecting through a wetland cluster comprised only of VC wetlands. In cases in which a wetland was subsumed by adjacent stream-connected wetlands or a stream-connected lake, the wetland was considered to be “connected.” Connection through an SI wetland was identified when an SI wetland co-occurred within a continuous Landsat polygon with VC wetlands. As lakes can occur as SI, VC or NCO features, connection through a lake was not specifically distinguished in this analysis. References to wetlands, therefore, could include water bodies specified by NWI as lakes or ponds.

### Euclidean and flowpath distance

Euclidean and flowpath distances to stream were calculated for each VC and NCO wetland. Although many factors may influence individual wetland hydrology (e.g., water retention capacity, topography, flow characteristics), simple parameters such as distance can be appealing to decision-makers, who need “bright line boundaries” between policy categories (Alexander 2015). It is therefore worthwhile to consider correlations with easily-measured structural parameters. Euclidean distances were calculated from the nearest edge of the wetland to the edge of the stream buffer. Flowpath distance for each wetland to stream were derived using the USGS NED 10 m resolution (Gesch et al. 2002). The buffered stream layer was converted to raster, overlaid onto the DEMs and assigned a no-data value so that topographic flows would end at the stream buffer edge. The DEMs were filled so that flow direction for all elevation cells could be routed to the stream edge. Stream raster layers were converted to “0” values to allow for complete flow length and flow accumulation measurements and flow length was calculated from each wetland’s “spill point,” identified as the point on each wetland perimeter with the greatest flow accumulation value (Blaszczynski 1997). The distance analysis was performed using Esri ArcGIS 10.2 (Esri 2013) and Arc Hydro (Maidment 2002). Cumulative distribution functions, ANOVA and Tukey HSD post hoc tests, using log-transformed data, were run in R to investigate variation in mean distance between ecoregions (R Core Team 2014). Log-mean distances and 95% confidence intervals were back-transformed to the original units (geometric means) when reported. In addition to tests of statistical significance, which are influenced by sample size, standardized mean differences (effect size, Cohen’s d) among ecoregions were compared to thresholds in Cohen (1988) to interpret the magnitude of effects in pairwise comparisons of mean wetland distance to stream.

### Variable importance assessment

Multiple regression and analysis of relative variable importance were used to quantify the contribution of the selected landscape variables to observed spatial variation in the abundance of derived wetland classes (VC, SI, and NCO). Regression assumptions were tested (R package car) (Fox and Weisberg 2011) and a Box-Cox power transformation (R package MASS, Venables and Ripley 2002) was applied to each of the dependent variables to correct for non-linearity and non-random distribution of residuals.

Each of the dependent variables (SI, VC, and NCO abundance) was found to be highly spatially auto-correlated, using Moran’s I (SI, z-score = 12.7, p < 0.01, VC, z-score = 6.9, p < 0.01, NCO, z-score = 12.5, p < 0.01), violating the assumption of independence. To account for this, an autocovariate was added that represented the area-weighted neighborhood response values of contiguous HUC10 polygons. By including a spatial autocovariate (e.g., Dormann et al. 2007; Betts et al. 2009) in the regression model, we control for how much the response variable reflects response values of adjacent HUCs, before identifying additional significant explanatory variables. Adding an autocovariate transforms the linear predictor of a generalized linear model from its usual form,${\text{y}}\;{ = }\;{\text{X}}|\upbeta \;{ + }\;\varepsilon$, (1) to ${\text{y}}\;{ = }\;{\text{X}}\beta \;{ + }\;{\text{pA}}\;{ + }\;\varepsilon$ (2), where β is a vector of coefficients for intercept and explanatory variables X, p is the coefficient of the autocovariate A, and ε is the vector of random errors. For the models tested the inclusion of an autocovariate removed the effect of spatial autocorrelation on the residuals (SI, z-score = 1.9, p = 0.06, VC, z-score = 0.3, p = 0.8, NCO, z-score = −1.1, p = 0.3). Alternative methods to account for spatial autocorrelation were also tested (e.g., simultaneous autoregressive models) (R package, spdep) (Kissling and Carl 2008), but did not produce AIC values as low as when an autocovariate was used.

Multicollinearity was assessed using the regression collinearity diagnostics described by Belsley et al. (1980) and implemented in the R package perturb (Hendrickx 2012). Collinearity may affect parameter estimation when a condition index (CI) greater than 10 is associated with variance decomposition proportions (VDP) greater than 0.5 for two or more explanatory variables (Belsley 1991). For the models tested, independent variables representing maximum surface-water extent, change in surface water extent and areal wetland abundance were identified as highly correlated (CI 19 and VDP > 0.75). Change in surface-water extent (maximum—minimum) and areal wetland abundance were removed, as these variables were interpreted to be redundant with and less informative than maximum surface-water extent, and regression diagnostics including collinearity were re-run for the reduced models (CI < 7 for all models).

Given the exploratory nature of this analysis, we compared four approaches for quantifying the relative contribution of the explanatory variables. In the first, the sum of Akaike weights provided the ratio of the change in AICc for each linear model that includes a specific variable to the whole set of possible linear models (R package, MuMIn) (Barton 2012). In the second, a hierarchical partitioning algorithm (Chevan and Sutherland 1991) was applied to the root-mean-square “prediction” error for all possible models to produce the independent (I) and conjoined (J) contribution of each variable (R package, hier.part) (Walsh and MacNally 2003; Murray and Conner 2009). Third, random forests were used (500 trees) and variable importance was calculated as the change in node impurity (Gini importance) (R package, randomForest) (Liaw and Wiener 2015). Lastly we calculated conditional permutation variable importance derived from the cforest algorithm in R (500 trees), which is designed to reduce bias introduced by multicollinearity (R package, party) (Strobl et al. 2008; Hothorn et al. 2015). The normalized results are presented to allow for the comparison of results across multiple tests. All statistical analyses were completed in R (R Core Team 2014) and the Global Moran’s I tests were completed in ArcGIS 10.2.2 (ESRI 2013).

## Results

### Distance patterns of wetlands by class

**Fig. 4:**
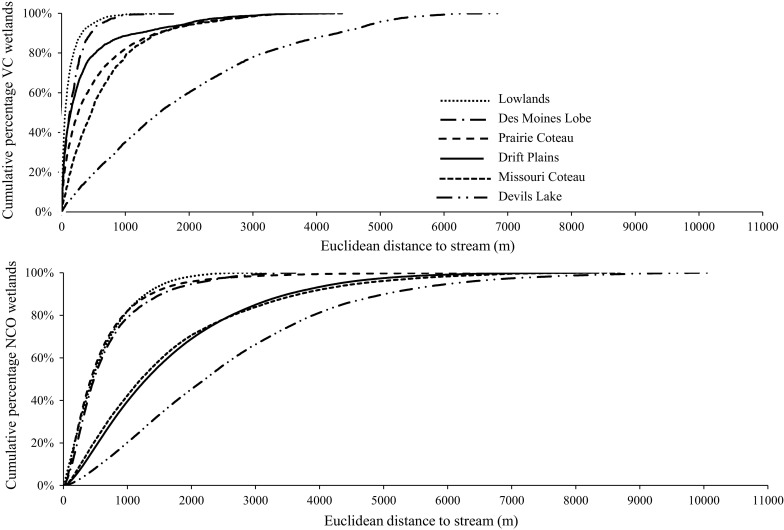
Cumulative distribution function of Euclidean distance to stream for VC (variably connected) wetlands (*top*) relative to NCO (no connection observed) wetlands (*bottom*), by ecoregion

The inclusion of several Landsat images from particularly wet years allowed us to identify a subset of wetlands that showed a variable connection to streams (Fig. 4). The percentage of wetlands classified as VC wetlands exceeded the percentage of SI wetlands in every ecoregion except the Lowlands (Table 5), and was almost double the percentage of SI wetlands across the entire study area. Wetlands classified as NCO, however, were the majority wetland class in all six ecoregions, ranging from 63.9% in the Des Moines Lobe to 92.4% in the Missouri Coteau (Table 5). Patterns in VC wetland distance to stream were evident, but substantially different between ecoregions (Fig. 4). VC wetlands occurred on average, closer to streams than NCO wetlands in each of the six ecoregions. However, the average distance an NCO wetland occurred in relation to a stream was smaller in the Lowlands, Des Moines Lobe and Prairie Coteau than the average distance that a VC wetland occurred from a stream in the Devils Lake ecoregion (Table 6; Fig. 4). Because Devils Lake water level expanded dramatically during periods of deluge, wetlands, previously long distances from the lake edge were subsumed, and therefore became part of a stream-connected lake. The distance over which this occurred was long relative to the other ecoregions, with a mean Euclidean distance of wetland to stream for VC wetlands of 1104 m (mean flowpath distance = 2466 m) (Table 6; Fig. 4). Flowpath distance showed similar between-ecoregion patterns but were much greater relative to Euclidean distances (123–203% greater for VC wetlands and 141–199% greater for NCO wetlands, by ecoregion). Effect sizes were insensitive to distance measure (Euclidean vs. flowpath), so effect size results for Euclidean and flowpath were averaged by wetland class (NCO and VC) (Table 7). The magnitude of effect for distance to stream was largest for comparisons of other ecoregions with Devils Lake. Effect sizes for distance to stream were negligible between the Missouri Coteau and the Drift Plains, and between the Des Moines Lobe and Prairie Coteau, for both NCO and VC wetlands. Effect size of distance to stream was also negligible for NCO wetlands, but not for VC wetlands, between the Lowland, Des Moines Lobe and Prairie Coteau ecoregions (Table 7).

**Table 5:** Wetland abundance (count), relative distribution and mean size (plus and minus standard error) by wetland class (SI = stream intersect, VC = variably connected, NCO = no connection observed) and ecoregion

Ecoregion	SI wetlands (%)	VC wetlands (%)	NCO wetlands (%)	SI wetland abund. (per km^2^)	VC wetland abund. (per km^2^)	NCO wetland abund. (per km^2^)	SI wetland size (ha)	VC wetland size (ha)	NCO wetland size (ha)
Lowland	17	13.8	69.2	1.1	0.9	4.3	2.8 ± 0.6	0.6 ± 0.03	0.3 ± 0.01
Des Moines Lobe	16.6	18.7	63.9	0.4	0.5	1.5	10.7 ± 2.1	1.1 ± 0.07	0.9 ± 0.04
Prairie Coteau	11.6	13.2	74.7	0.8	1.0	5.2	8.6 ± 0.9	1.6 ± 0.1	0.6 ± 0.01
Missouri Coteau	3.1	4.4	92.4	0.3	0.5	10.3	10.0 ± 1.6	3.6 ± 0.5	0.8 ± 0.02
Drift Plains	3.3	9.5	85.2	0.4	1.1	9.9	5.8 ± 0.5	1.0 ± 0.05	0.5 ± 0.01
Devils Lake	0.7	14.8	82.3	0.1	2.9	15.1	79.3 ± 25.1	1.0 ± 0.06	0.5 ± 0.01
All	6.2	11.3	81.4	0.8	1.5	10.8	8.7 ± 0.7	1.9 ± 0.07	0.6 ± 0.01

**Table 6:** Comparison of Euclidean and flowpath distance to stream for VC (variably connected) and NCO (no connection observed) wetland classes by ecoregion

Ecoregion	VC wetland Euclidean distance to stream (m)^a^	VC wetland flowpath distance to stream (m)^b^ ^1^	NCO wetland Euclidean distance to stream (m)^c^ ^2^	NCO wetland flowpath distance to stream (m)^d^ ^3^	VC wetland distance increase (%)	NCO wetland distance increase (%)
Lowland	67.9^1^ (66.7, 69.2, n = 7597)	205.8^1^ (202.4, 209.4, n = 7597)	352.0^1^ (349.8, 354.1, n = 38,199)	943.3^1^ (937.1, 949.5, n = 38,199)	203.0	168.0
Des Moines Lobe	128.2^2^ (125.2, 131.3, n = 3830)	346.4^2^ (338.5, 354.5, n = 3830)	426.9^2^ (422.9, 430.9, n = 12,542)	1050.7^2^ (1039.9, 1061.7, n = 12,542)	170.2	146.1
Prairie Coteau	189.2^3^ (186.4, 192.0, n = 11,653)	442.7^3^ (436.5, 449.0, n = 11,653)	376.9^3^ (375.2, 378.5, n = 63,305)	908.6^3^ (904.3, 912.9, n = 63,305)	134.0	141.1
Missouri Coteau	281.0^4^ (273.0, 288.9, n = 2631)	644.0^4^ (626.9, 661.7, n = 2631)	1042.8^4^ (1038.2, 1047.5, n = 54,133)	2929.1^4^ (2913.9, 2944.4, n = 54,133)	129.2	180.9
Drift Plains	302.8^4^ (299.5, 306.1, n = 20,610)	762.5^5^ (754.0, 771.1, n = 20,610)	1115.1^5^ (1112.5, 1117.6, n = 187,585)	2863.6^4^ (2856.1, 2871.2,n = 187,585)	151.8	156.8
Devils Lake	1103.75 (1091.7),1115.8, n = 12,752)	2465.9^6^ (2436.3, 2495.9, n = 12,752)	1844.9^6^ (1838.6, 1851.3, n = 66,827)	4702.9^5^ (4684.2, 4721.7, n = 66,827)	123.4	154.9
All	283.8 (281.8, 285.8, n = 59,073)	708.2 (703.2, 713.2, n = 59,073)	891.1 (889.5, 892.7, n = 422,591)	2298.1 (2293.5, 2302.7, n = 422,591)	149.5	198.7

Columns 1–4: Geometric mean distance (and 95% confidence interval) by class and ecoregion. Superscripts indicate significant (p ≪ 0.05) differences in pairwise comparisons of ecoregion means using Tukey’s honest significant difference (HSD) tests of log-transformed distance. Columns 5–6: Percentage increase in mean wetland distance to stream using flowpath versus Euclidean distance

Differences between ecoregions were significant for all categories (p < 0.01) using ANOVA (^a^F = 3891, ^b^ ^1^F = 3051, ^c^ ^2^F = 25,500, ^d^ ^3^F = 19,309)

**Table 7:** Standardized mean differences in VC (variably connected) and NCO (no connection observed) wetland distance to stream

	Lowland		Des Moines Lobe		Prairie Coteau		Missouri Coteau		Drift Plains	
	VC	NCO	VC	NCO	VC	NCO	VC	NCO	VC	NCO
Des Moines Lobe	0.36.	0.13								
Prairie Coteau	0.51*	0.04	0.15	0.13						
Missouri Coteau	0.74*	0.91**	0.41.	0.81**	0.24.	0.93**				
Drift Plains	0.84**	1.03**	0.49.	0.91**	0.34.	1.02**	0.11	0.04		
Devils Lake	1.72***	1.51**^*^	1.41***	1.48***	1.17^**^	1.52***	0.98**	0.51*	0.76*	0.48*

Superscripts indicate effect size thresholds for pairwise comparisons of ecoregion means reported in Table 6. Effect sizes were not sensitive to distance measure so results for Euclidean and flowpath distance measures were averaged within wetland classes (VC, NCO)

Effect size: Very large 1.3 ‘***’ Large 0.8 ‘**’ Medium 0.5 ‘*’ Small 0.2 ‘.’ (Cohen 1988)

### Mechanism of connection for VC wetlands

**Fig. 5:**
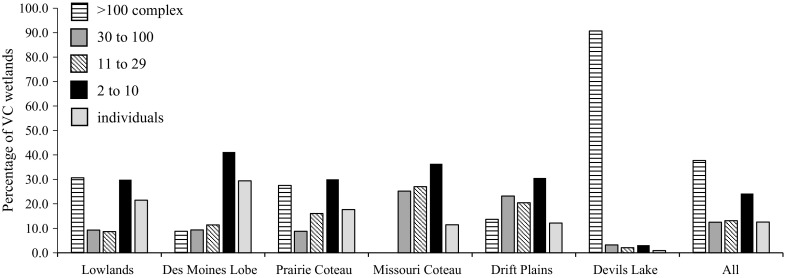
The distribution of variably connected (VC) wetlands (saturated) by complex size and ecoregion under deluge conditions, or the two wettest dates per time series

SI wetlands were found to play an important role in merging or consolidating VC wetlands (40–80% across ecoregions) with streams (Table 8). The merging (or subsuming) of VC wetlands with one another and merging with streams, in a stepping-stone or consolidation manner, independent of SI wetlands also played a substantial role, connecting approximately 20–30% of VC wetlands in deluge conditions, depending on the ecoregion (Table 8). The importance of wetlands merging individually to streams (i.e., no stepping-stone activity), showed variable importance, but was particularly important in the Des Moines Lobe which contains the Minnesota River (connected almost 30% of the VC wetlands in this ecoregion) (Table 8). Wetland clusters (surficially connected or consolidated wetlands or the co-occurrence of more than one NWI wetland within a single Landsat surface-water extent polygon) of multiple size classes were found to be important in connecting VC wetlands to streams (Fig. 3). Across all ecoregions under deluge conditions, connected wetland clusters containing over 100 wetlands were found to contain the majority of the VC wetlands (37.7%), however smaller wetland clusters (e.g., two to ten wetlands) were also found to be critical, containing 24.2% of VC wetlands (Fig. 5). The frequency of different sized wetland clusters also varied substantially between ecoregions. In the Des Moines Lobe, for example, most VC wetlands connected either individually (29.4%), or through small clusters (2–10 VC wetlands) (41.2%), while in Devils Lake ecoregion, 90.7% of the VC wetlands connected via wetland clusters with more than 100 VC wetlands in the cluster (i.e., the expansion of Devils Lake) (Fig. 5).

**Table 8:** Mechanism of connection for VC (variably connected) wetlands under deluge conditions, defined as the two wettest images per path/row

Ecoregion	Merging with SI wetlands (%)	Expansion of individual wetlands (%)	Expansion and merging with other VC wetlands (%)
Lowland	46.0	21.5	32.5
Des Moines Lobe	44.7	29.4	25.9
Prairie Coteau	60.2	17.7	22.1
Missouri Coteau	65.9	11.4	22.7
Drift Plains	54.8	12.1	33.1
Devils Lake	82.2	0.9	16.9
All	60.4	12.5	27.1

Expansion of individual wetlands refers to those expanded wetlands that connect directly to the stream layer. Merging with SI (stream intersect) wetlands refers to wetlands merging in a stepping-stone fashion with the end member an SI wetland. Expansion and merging with other VC wetlands refers to a similar stepping-stone merging, but with the end member the stream layer

### Variable importance in explaining wetland class abundance

**Fig. 6:**
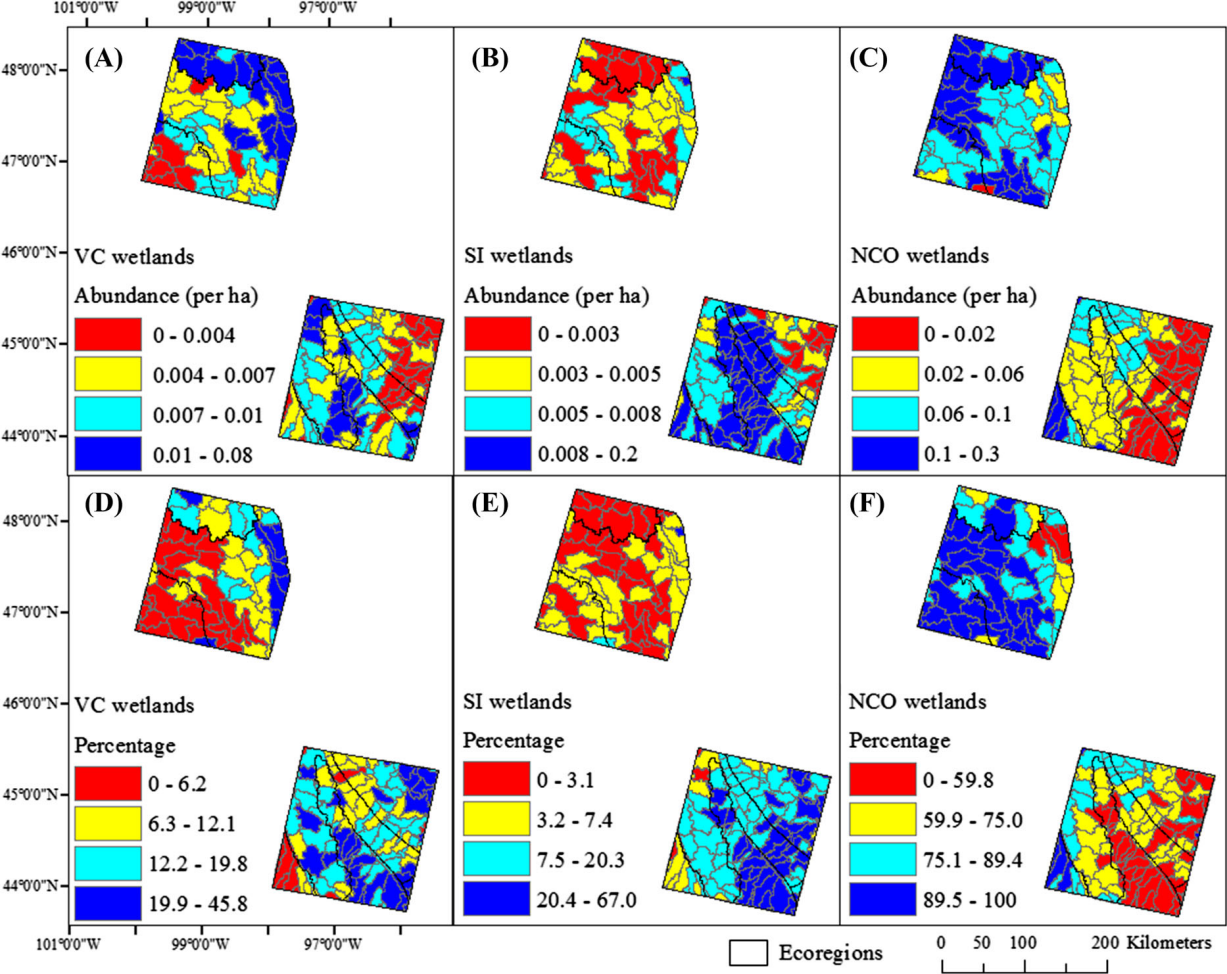
Differences in the spatial pattern of VC (variably connected), SI (stream intersect), and NCO (no connection observed) wetland abundance by wetlands per ha (**a**, **b**, **c**) and relative percentage (**d**, **e**, **f**), where percentage is calculated from the sum of VC, SI and NCO wetlands. Category divisions represent quantiles

The abundance of wetland types (SI, VC, NCO) showed strong spatial patterns (Fig. 6). After controlling for the spatial autocorrelation of wetland abundance, increases in the abundance of SI wetlands were best explained by increases in stream density and smaller mean distances between wetlands and streams. These two variables were consistently important across all four approaches used to evaluate relative variable importance (Table 9). The abundance of NCO wetlands were most highly correlated with wetland density (Table 9). However, because most wetlands across the study area were classified as NCO wetlands (Table 5), this explanatory variable was seen as uninformative. Similarly, wetland to wetland distance was highly correlated with wetland density (R = −0.95) and therefore also uninformative (Table 10). Variability in NCO wetland abundance was best explained by the percent of land that was drained by anthropogenic means. NCO wetland abundance decreased as more of each HUC was artificially drained. This explanatory variable was consistently important across all four approaches. Stream density and wetland to stream distance were also ranked as important in more than one approach. NCO wetland abundance increased with lower stream density and larger mean wetland to stream distances. Lastly, total wetland density and wetland to wetland distance were identified as the most consistently important variables to explain variability in VC wetland abundance after controlling for spatial autocorrelation. Meanwhile, maximum surface water extent also ranked as an important variable in VC abundance by more than one approach. VC wetland abundance increased with more wetlands, located in close proximity to one another, and large maximum surface water extents during wet periods (Fig. 5).

**Table 9:** The relative importance of explanatory variables in explaining spatial variation in the abundance of SI (stream intersect), VC (variably connected) and NCO (no connection observed) wetland classes

SI wetlands	Spearman rank correlation	Sum of “Akaike weights” (all models) (ratio)*	AWsum (%)	Hierarchical partitioning (I + J)*	Hierarchical partitioning (I %)	Gini importance (randomForest)	Gini importance (%)	Conditional Permutation (cforest) (CP)	CP (%)
Autocovariate	0.80*	1	11.2	−1.76E−01	19.8	5.17E−02	31.3	2.72E−04	54.5
**Stream density**	0.72*	1	**11.2**	−1.24E−01	**17.5**	3.05E−02	**18.5**	6.33E−05	**12.7**
**Wetland to stream distance**	−0.64*	1	**11.2**	−8.76E−02	**13.6**	1.89E−02	**11.5**	5.96E−05	**12.0**
Lake abundance	−0.31*	1	**11.2**	−1.65E−02	5.5	2.64E−03	1.6	2.05E−07	0.0
Lake areal abundance	−0.25	0.39	4.4	−8.56E−03	4.5	2.92E−03	1.8	1.06E−06	0.2
Max surface-water extent	−0.51*	0.99	**11.1**	−5.44E−02	8.3	6.85E−03	4.1	5.49E−06	1.1
Change in surface-water extent	−0.45*	Excluded	Excluded	Excluded	Excluded	4.64E−03	2.8	1.76E−06	0.4
Wetland to wetland distance	0.21	1	**11.2**	−2.80E−03	7.3	6.49E−03	3.9	2.33E−05	4.7
Total wetland density	−0.10	0.63	7.1	−3.35E−03	5.4	8.27E−03	5.0	7.28E−06	1.5
Wetland areal abundance	−0.32*	Excluded	Excluded	Excluded	Excluded	3.37E−03	2.0	1.73E−06	0.3
Percent drained	−0.26	1	**11.2**	−2.29E−02	6.0	6.60E−03	4.0	2.69E−05	5.4
Elevation coeffic. of var.	0.47*	0.64	7.2	−2.96E−02	5.9	8.61E−03	5.2	1.31E−05	2.6
Melton ruggedness number	0.50*	0.28	3.1	−3.52E−02	6.2	1.29E−02	7.8	2.27E−05	4.6

VC wetlands	Spearman rank correlation	Sum of “Akaike weights” (all models) (AWsum)*	AWsum (%)	Hierarchical partitioning (I + J)*	Hierarchical partitioning (I %)	Gini importance (randomForest)	Gini importance (%)	Conditional Permutation (cforest) (CP)	CP (%)
Autocovariate	0.73*	1	14.4	−2.51E−01	22.3	1.72E−01	32.2	1.4E−03	77.8
Stream density	−0.04	0.98	**14.1**	−1.31E−03	7.7	2.39E−02	4.5	3.9E−05	2.2
Wetland to stream distance	0.16	0.72	10.4	−1.94E−02	7.0	2.33E−02	4.3	2.2E−05	1.3
Lake abundance	0.25	0.26	3.7	−1.91E−02	7.1	1.31E−02	2.4	−6.3E−07	0.0
Lake areal abundance	0.08	0.32	4.6	−4.02E−05	6.4	1.30E−02	2.4	1.4E−05	0.8
*Max surface-water extent*	0.22	1	**14.4**	−3.48E−02	**8.5**	1.78E−02	3.3	1.9E−05	1.1
Change in surface-water extent	0.29*	Excluded	Excluded	Excluded	Excluded	2.73E−02	5.1	3.7E−05	2.1
Wetland to wetland distance	−0.40*	0.99	**14.3**	−1.14E−01	**12.8**	6.21E−02	**11.6**	1.3E−04	**7.1**
Total wetland density	0.42*	0.58	8.4	−8.09E−02	**11.0**	8.52E−02	**15.9**	9.2E−05	*5.2*
Wetland areal abundance	0.30*	Excluded	Excluded	Excluded	Excluded	1.64E−02	3.1	6.7E−06	0.4
Percent drained	−0.18	0.31	4.5	−7.68E−02	4.1	5.43E−02	**10.1**	4.8E−05	2.7
Elevation coeffic. of var.	0.11	0.32	4.6	−3.94E−04	6.4	1.49E−02	2.8	−2.5E−06	−0.1
Melton ruggedness number	−0.01	0.46	6.6	−5.00E−04	6.4	1.22E−02	2.3	−3.0E−06	−0.2

NCO Wetlands	Spearman rank correlation	Sum of “Akaike weights” (all models) (AWsum)*	AWsum (%)	Hierarchical partitioning (I + J)*	Hierarchical partitioning (I %)	Gini importance (randomForest)	Gini importance (%)	Conditional Permutation (cforest) (CP)	CP (%)
Autocovariate	0.85*	1	16	−7.77E−01	45.4	8.04E−01	34	6.27E−03	81.2
*Stream density*	−0.57*	0.99	**16**	−2.31E−01	**10.8**	1.50E−01	6	2.29E−04	3.0
*Wetland to stream distance*	0.69*	0.26	4	−2.94E−01	**9.9**	3.73E−01	**16**	1.85E−04	2.4
Lake abundance	0.47*	0.46	7	−1.36E−01	4.2	1.16E−01	5	1.38E−04	1.8
Lake areal abundance	0.00	0.95	**15**	−7.79E−03	1.4	6.93E−02	3	7.05E−05	0.9
Max surface-water extent	0.35*	0.26	4	−9.71E−02	2.7	6.12E−02	3	2.64E−05	0.3
Change in surface-water extent	0.55*	Excluded	Excluded	Excluded	Excluded	1.20E−01	5	2.11E−05	0.3
Wetland to wetland distance	−0.86*	Excluded	Excluded	Excluded	Excluded	Excluded	Excluded	Excluded	Excluded
Total wetland density	0.90*	Excluded	Excluded	Excluded	Excluded	Excluded	Excluded	Excluded	Excluded
Wetland areal abundance	0.51*	Excluded	Excluded	Excluded	Excluded	2.24E−01	10	1.30E−04	1.7
**Percent drained**	−0.55*	1	**16**	−3.75E−01	**21.6**	3.14E−01	**13**	5.87E−04	**7.6**
Elevation coeffic. of var.	0.04	0.98	**15**	−6.32E−04	2.3	5.88E−02	3	2.43E−05	0.3
Melton ruggedness number	0.04	0.45	7	−2.34E−03	1.6	6.05E−02	3	4.52E−05	0.6

Individual Spearman rank correlations are also shown with the Bonferroni correction applied. Significant correlations are starred. “Important” variables within each approach are identified using natural breaks in the normalized results (italic font). Variables identified as “important” in most approaches are in bold font. Variables identified as “important” in some of the approaches are in italic font. This notation is meant only to highlight results and does not represent an objective analysis of the results

*n = 1024, 1024 and 256 models for SI, VC and NCO, respectively, for Akaike weights and hierarchical partitioning approaches. The summed independent (I) and conjoined (J) contribution of each variable is shown for the hierarchical partitioning approach

**Table 10:** Spearman rank correlations between the independent explanatory variables (n = 155)

Independent Variables	Stream density	Wetland to stream distance	Lake abund.	Lake areal abund.	Max surface-water extent	Change in surface-water extent	Wetland to wetland distance	Total wetland density	Wetland areal abund.	Percent drained	Elevation coeffic. of var.	Melton ruggedness number
Stream density	1	−0.88*	−0.67*	−0.35*	−0.69*	−0.74*	0.62*	−0.54*	−0.65*	0.19	0.50*	0.44*
Wetland to stream distance	−0.88*	1	0.65*	0.29*	0.70*	0.78*	−0.76*	0.70*	0.71*	−0.29*	−0.24	−0.34*
Lake abundance	−0.67*	0.65*	1	0.55*	0.77*	0.77*	−0.57*	0.46*	0.83*	−0.18	−0.27	−0.24
Lake areal abundance	−0.35	0.29	0.55*	1	0.62*	0.42*	−0.14	−0.03	0.66*	0.00	−0.23	−0.38*
Max surface-water extent	−0.69*	0.70*	0.77*	0.62*	1	0.91*	−0.50*	0.37*	0.88*	−0.07	−0.30*	−0.45*
Change in surface-water extent	−0.74*	0.78*	0.77*	0.42*	0.91*	1	−0.67*	0.60*	0.80*	−0.23	−0.31*	−0.41*
Wetland to wetland distance	0.62*	−0.76*	−0.57*	−0.14	−0.50*	−0.67*	1	−0.95*	−0.66*	0.48*	−0.01	0.04
Total wetland density	−0.54*	0.70*	0.46*	−0.03	0.37*	0.60*	−0.95*	1	0.52*	−0.57*	0.05	0.04
Wetland areal abundance	−0.65*	0.71*	0.83*	0.66*	0.88*	0.80*	−0.66*	0.52*	1	−0.29*	−0.16	−0.29*
Percent drained	0.19	−0.29*	−0.18	0.00	−0.07	−0.23	0.48*	−0.57*	−0.29*	1	−0.12	−0.15
Elevation coeffic. of var.	0.50*	−0.24	−0.27	−0.23	−0.30*	−0.31*	−0.01	0.05	−0.16	−0.12	1	0.69*
Melton ruggedness number	0.44*	−0.34*	−0.24	−0.38*	−0.45*	−0.41*	0.04	0.04	−0.29*	−0.15	0.69*	1

Significant correlations are determined using the Bonferroni correction and are marked with asterisks

## Discussion

This study provides one of the first landscape-scale efforts to explore spatial patterns and landscape drivers of dynamic surface-water connections between depressional wetlands and streams in the PPR. These VC wetlands were found to connect to streams predominately through merging with and being subsumed by other wetland features. Both small (2–10) and large (>100) wetland clusters (or complexes of surficially connected or consolidated wetlands) were common across the study area. The consolidation of wetlands was particularly common around lake features, many of which occur in open, flat basins in which excess water can result in 100% to almost 600% increases in surface-water extent (Vanderhoof and Alexander 2015) (Fig. 6). Initial rises in lake levels may merge wetlands with lakes, but wetlands may still retain wetland vegetation and function. However, as lake levels continue to rise, merged wetlands are completely subsumed by lakes and no longer function as independent depressional wetlands (Mortsch 1998). Features were observed to expand and contract in response to variable wetness conditions, connecting and disconnecting lakes, streams and wetlands. Previous work in the PPR documented variability in wetland-to-wetland and wetland-to-stream connectivity as surface water merges in low relief areas and/or wetlands fill and spill (Leibowitz and Vining 2003; Kahara et al. 2009; Shaw et al. 2013; Vanderhoof et al. 2016), and sought to predict connectivity based on storage capacity and spill point elevation (Huang et al. 2011b), temporal changes in surface-water extent (Rover et al. 2011), and wetland vegetation and water chemistry (Cook and Hauer 2007). This study sought to move from the prediction of connections for individual wetlands to explaining variability in the abundance of such surface-water connections on a landscape scale.

The probability of hydrologic connectivity has been most commonly linked to the proximity or distance between depressional wetlands and streams (Tiner 2003; Kahara et al. 2009; Lang et al. 2012). Yet this study found that substantial variation in the mean Euclidean and flowpath distance to stream for VC and NCO wetlands between ecoregions makes it extremely problematic to identify VC wetlands based on distance alone. For example within 400 m of a stream on the Des Moines Lobe, 78% of the VC wetlands were connected, while the Drift Plains had only 52% of the VC wetlands connected at that same distance. Consequently while mean distance to stream emerged as an important variable in explaining the abundance of SI and NCO variables, it was not ranked as important in explaining the abundance of VC wetlands. Instead, for VC wetlands, wetland arrangement (wetland to wetland distance), as well as the temporal dynamics of surface-water expansion, also need to be considered. Additionally, in landscapes with little relief, flowpath distance from a fixed spill point to a fixed stream entry point may be less relevant. Surface flows connecting wetlands to streams in this area may not follow a single, theoretical flowpath, but instead are likely to expand and spread across the flat surface as excess water accumulates in a catchment.

The variables considered in the models represent several different factors in determining landscape-scale connectivity including (1) wetland abundance, (2) wetland arrangement (distance variables), (3) the availability of surface water connections (stream and lake abundance, surface water extent), and (4) potential influences on water accumulation and flow (topography and land use variables). However, across the PPR, variability within and between these variables is intrinsically tied to variability in landscape age (since last glacial retreat) and corresponding drainage development across the region (Ahnert 1996). The last maximum glacial extent (the Wisconsin glacier) diverged around the Lowlands ecoregion, leaving the older landscape (>20,000 BP) with a well-developed drainage network (Clayton and Moran 1982). In contrast, the Wisconsin glacier retreated from the Missouri Coteau and Drift Plains ecoregions by 11,300 BP, meaning the drainage system is still developing in these ecoregions. In ecoregions with low drainage development, surface water is being stored in glacially formed depressions (Winter and Rosenberry 1998; Stokes et al. 2007), resulting in an inverse relationship between stream density and surface-water extent (Table 10). The drainage network in the PPR is also increasingly modified with the expansion of ditch networks and tile drainage in association with agricultural activities (McCauley et al. 2015). Ditches, pipes and field tiles can increase connectivity between waterbody features, however, both filling wetlands with soil and lowering the water table through increased water withdrawal can decrease expected surface-water connectivity (DeLaney 1995; Blann et al. 2009; McCauley et al. 2015). Our finding regarding the importance of predicted anthropogenic drainage may be related to the relation between land use and wetland connectivity and wetland loss (Miller et al. 2009; Van Meter and Basu 2015). These potential interrelations merit further study.

It is critical to note that the aim of this analysis was not to document all surface-water connections, recognizing limitations of our input datasets, but instead, to characterize spatial patterns for a subset of wetlands that merge with a stream over a wide range of wetness conditions and a relatively large study area. A complete analysis of wetland-to-stream connectivity would also need to consider narrow and temporary (e.g., in response to rain events and peak snow melt conditions) surface connections, groundwater connections, as well as chemical and biological connections (U.S. EPA 2015). This analysis allowed us to identify regionally relevant parameters that can provide a preliminary means to explain variability in the abundance of wetlands that affect streamflow and are subject to regulatory programs. Patterns in VC wetland abundance, for example, demonstrate that wetland abundance and arrangement in combination with expanding surface-water extent provides important opportunities for wetlands to merge with streams, a finding consistent with related literature. Limitations of this study are potential bias due to unmeasured variables and the glacial history of the landscape, which may complicate efforts to apply these variables to different ecoregions.

Further, patterns in the mechanism of connection show that in addition to SI wetlands, depressional wetlands and open waters can play critical roles in moving surface water across the landscape. These findings are particularly relevant to floodplains, permafrost landscapes and formerly glaciated landscapes that often exhibit low topographic gradients, low rates of infiltration, and low stream density. Runoff events in these landscapes rarely satisfy the threshold surface storage volume so that excess surface water (precipitation inputs exceeding soil infiltration and evapotranspiration) tends to accumulate instead of leaving the watershed as stream discharge (Hamilton et al. 2004; Yao et al. 2007; Aragón et al. 2011; Kuppel et al. 2015), leading to wetland consolidation and surface-water connections.

## Conclusion

Variably connected wetlands represent a critical subset of wetlands that may appear disconnected from streams under dry or average conditions, but exchange water and materials with streams under wetter conditions. Substantial spatial variation in the distance over which wetlands merged (or did not merge) with streams demonstrated that any characterization of connectivity based on proximity would need to be highly regionalized. More consistent across ecoregions was the mechanism of wetlands connecting to streams through wetland consolidation or wetland clusters, in particular those clusters containing an SI wetland. We documented substantial spatial variation in the relative abundance of SI, VC and NCO wetlands. The variation in abundance was best explained by different variables for each class of wetlands. However, wetland spatial arrangement, both between wetlands and between wetlands and streams, as well as the availability of surface-water connections, whether through higher stream density or larger surface-water expansion, were identified as critical to explaining the abundance of or lack of connections between wetlands and streams. Understanding the mechanisms through which wetlands merge with streams, and the spatial patterns that drive the abundance of VC wetlands in the PPR are crucial to understanding their influence on downstream waters, as well as accurately predicting flood events and the consequences of climate change on surface-water distribution, movement, and connectivity.
